# Use of Finite Elements in the Training of a Neural Network for the Modeling of a Soft Robot

**DOI:** 10.3390/biomimetics8010056

**Published:** 2023-01-28

**Authors:** Silvia Terrile, Andrea López, Antonio Barrientos

**Affiliations:** 1 Centre for Automation and Robotics (CAR), Universidad Politécnica de Madrid—Consejo Superior de Investigaciones Científicas, 28006 Madrid, Spain; 2 Departamento de Ingeniería Eléctrica, Electrónica, de Comunicaciones y Sistemas, University of Oviedo, 33203 Gijón, Spain

**Keywords:** soft robotics, finite elements method, SMA, neural networks

## Abstract

Soft bioinspired manipulators have a theoretically infinite number of degrees of freedom, providing considerable advantages. However, their control is very complex, making it challenging to model the elastic elements that define their structure. Finite elements (FEA) can provide a model with sufficient accuracy but are inadequate for real-time use. In this context, Machine Learning (ML) is postulated as an option, both for robot modeling and for its control, but it requires a very high number of experiments to train the model. A linked combination of both options (FEA and ML) can be an approach to the solution. This work presents the implementation of a real robot made up of three flexible modules and actuated with SMA (shape memory alloy) springs, the development of its model through finite elements, its use to adjust a neural network, and the results obtained.

## 1. Introduction

Soft robotics is a branch of robotics characterized by the use of sufficiently soft materials to make robots able to adapt their shape to the environment and interact more safely with it. Among the different applications that can be found, it is worth highlighting the soft bioinspired manipulators. These have a theoretically infinite number of degrees of freedom and may be suitable for those tasks in which higher dexterity is needed from the robotic arm (inspections, unstructured environments, and medical applications) [1].

Although the advantages provided by soft manipulators are considerable, their control is very complex. The elastic effects of the materials that compose them make it impossible to find an analytical model that relates the shape adopted by the manipulator as a whole [2,3] and, in particular, the location of its end with the movements of its actuators [4]. In addition to the difficulty of modeling the elastic elements that define the robot’s structure, there is often difficulty associated with the model of some actuators, as in the case of those based on shape memory alloys (SMA). As an alternative, computational methods, such as finite elements (FEA), can provide a model with sufficient accuracy [5,6,7] but are inadequate for real-time control. In this context, machine learning (ML) is postulated as a promising option for modeling the robot, actuator, and control [8,9].

Nevertheless, for its materialization, it requires a very high number of experiments to train the model, making this process, which implies the movement of the real robot, very inefficient. This work presents a chained combination of both options (FEA and ML) [10], allowing an efficient solution approach. First, an FEA model of adequate precision can be used to train a model based on a neural network that, once adjusted, will serve as a model to be used in real-time for robot control. It allows the robot’s end effector to be sent to a destination without knowing how each segment must move to reach the final position. For this, it is necessary to carry out the robot’s kinematic modeling and know the relationship between the actions of the actuators and the curvature of the resulting arc. This information will be used to feed a neural network and perform control.

**Figure 1:**
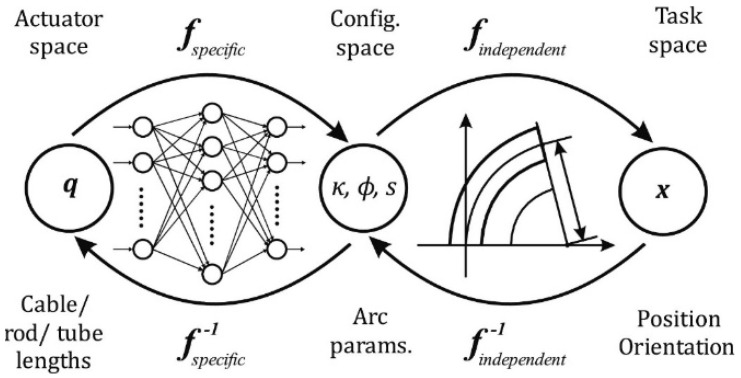
Mapping between spaces to define the kinematics of a constant curvature robot. Adapted from [4].

Three techniques have been used to carry out this work: segmental constant curvature kinematics (PCC), finite element analysis (FEA), and artificial neural networks (ANN). The starting point was the mapping proposed by [4] in the kinematics of constant curvature, whose scheme is presented in Figure 1.

Robot-independent mapping can be realized by employing different solutions [2] such as Denavit-Hartenberg parameters (such as in this case) [11,12], arc geometry [13,14], differential geometry [15,16], exponential coordinates [17] or integral representation [18].

The PCC approach simplifies kinematic modeling, and it is a good option when robots present low speed and mass since it is possible to ignore inertia effects [19,20]. In addition, PCC allows working with modular kinematic transformations that can be decomposed into two separate mappings: one from actuator space to configuration space and one from configuration space to task space.

The first mapping involves the transformation of the actuator space *q*, towards the configuration space whose parameters *K*, ∅, *S* describe the arcs of constant curvature. The second mapping involves the transformation of the configuration space into the task space *x*, which represents the position and orientation of both the central arc (backbone) and the tip of the robot.

The actuator space *q* is represented by the variables that describe the action of the actuators (cables’ length for a robot powered by tendons or value of the pressures for a pneumatic one). This work models a three-segment soft robot developed at CAR (UPM-CISC) called Ruǎn. In it, the actuator space *q* consists of the variables that describe the action of the SMA springs. These variables are mapped to those in the configuration space (*K*, ∅, *S*) through *f_specific_* mapping, which is highly dependent on the specific robot. In contrast, the second *f_independent_* mapping, between the configuration space and the task space (*x*), is not robot-dependent, which means that it is the same for all robots that meet the requirements of the PCC formalism.

Consequently, the direct kinematic transformation can be expressed as:

(1) $$q\;\overset{f_{specific}}{\to }\;K,\;\emptyset ,\;S\;\overset{f_{1}}{\to }\;\theta ,\;d\;\overset{f_{D-H\;}}{\to }\;x\;$$

The *f_independent_* mapping decomposes into two mappings: the first binds the arc parameters and the Denavit–Hartenberg (*D*–*H*) parameters, and the second binds the *D*–*H* parameters to the task space variables *x*. Inverse kinematic mappings $f^{-1}$ can be used for robot motion control.

In this case, the *f_independent_* is derived using PCC kinematic relations, as already mentioned, while the *f_specific_* is obtained from the finite elements (FEA). The main reasons are the complexity of the analytical calculations and the large number of experiments required to obtain them experimentally. Finite element simulation accurately represents physical robot behavior despite its complex structure. However, this method is computationally expensive in terms of time and resources and cannot be used in real time. Therefore, in this work, the data generated offline by the FEA simulations are used to train an ANN that is used later in the real-time control.

**Figure 2:**
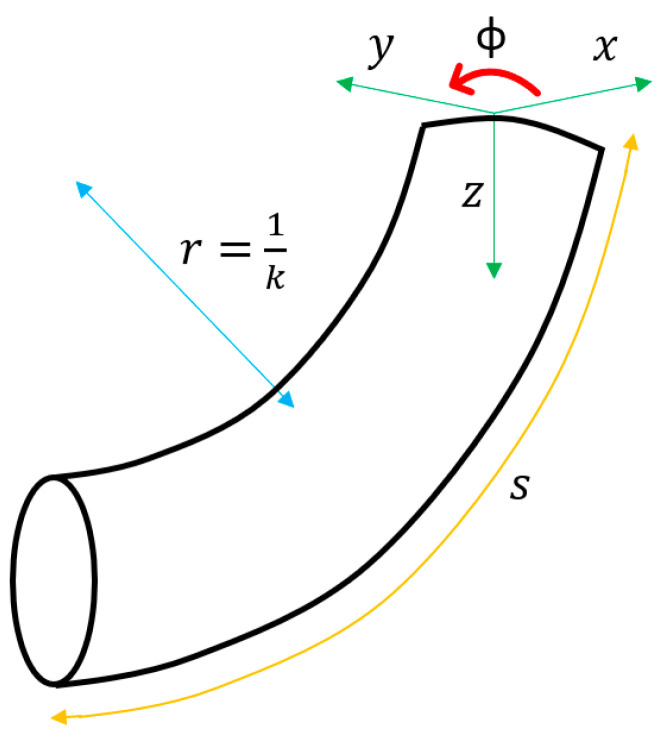
Kinematic model using the PCC method. Adapted from [10].

The PCC method was proposed by Hannan and Walker in 2000 [21]. Their work suggests that after activating the actuators, the curves generated in the robot body can be approximated as a series of arcs of constant curvature part tangent to each other. Likewise, these curves are described using the arc parameters *K*, ∅, *S* belonging to the robot’s configuration space, as illustrated in Figure 2.

As can be seen, *K* is the curvature (inverse of the circle’s radius that describes the arc), ∅ is the angle between the x-z plane and the plane that contains the arc, and *S* indicates the length of the arc.

**Figure 3:**
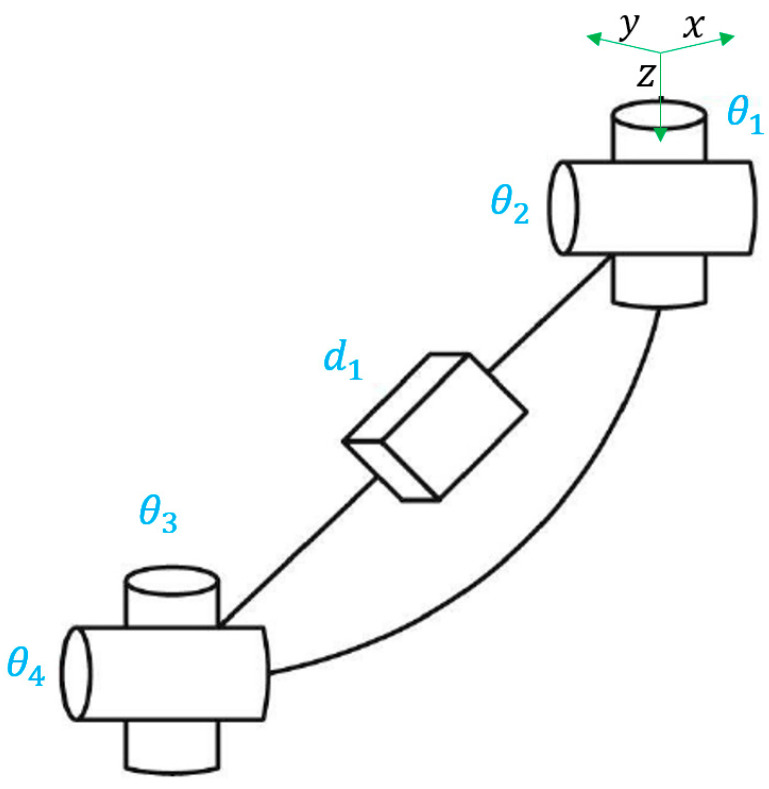
Hypothetical rigid link model of a flexible robot. Adapted from [10].

Webster and Jones showed in [4] that the segment could be represented as a rigid link with two rotational joints at the base, a prismatic joint in the middle, and two other rotational joints at the tip of the segment, as shown in Figure 3.

The parameters *d*1, *θ*1, *θ*2, *θ*3, *θ*4 correspond directly to the Denavit-Hartenberg parameters, so they can be used to define the homogeneous transformation matrix *T* (*d*, *θ*). Therefore, the arc parameters can be applied directly in the transformation matrix, being *T_PCC_* (*d*1, *K*, ∅, *S*). This matrix can be simplified since *θ*3 = *θ*2 and *θ*4 = *θ*1 + π, as shown by Jones and Walker [22]. So, the final *T_PCC_* representing *f_independent_* is:

(2) $$T_{pcc}=\left[\begin{array}{cccc} cos^{2}\emptyset \;\left(cosKS-1\right)+1 & sin\emptyset \;cos\emptyset \;\left(cosKS-1\right) & cos\emptyset \;sin\emptyset & \frac{cos\emptyset \left(1-cosKS\right)}{K}\\ sin\emptyset \;cos\emptyset \;\left(cosKS-1\right) & cos^{2}\emptyset \;\left(1-cosKS\right)+cosKS & sin\emptyset \;sinKS & \frac{sin\emptyset \;\left(1-cosKS\right)}{K}\\ -cos\emptyset \;sinKS & -sin\emptyset \;sinKS & cosKS & \frac{sinKS}{K}\\ 0 & 0 & 0 & 1 \end{array}\right]$$

**Figure 4:**
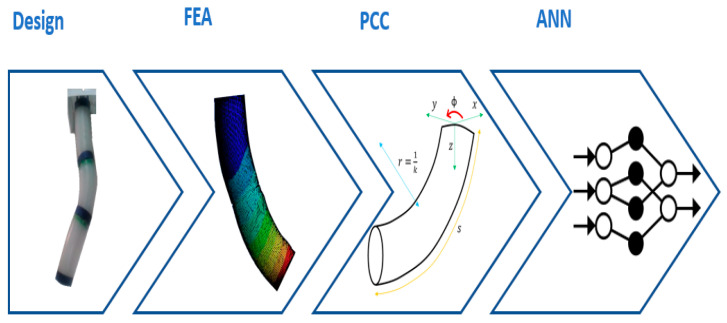
Kinematic modeling and design framework for soft robotic systems. Source: authors.

This work presents the implementation in a real robot consisting of three flexible modules actuated with SMA (shape memory alloy) springs, the development of its model through finite elements, and the use of this to adjust a neural network, as summarized in Figure 4. The aim is to test and validate the framework with a real soft robot.

## 2. Ruǎn Design, a Soft Continuous Manipulator

The continuous and soft Ruǎn manipulator is made up of three segments made of Platsil GEL-0030 silicone. Each segment is cylindrical and is independently actuated by three SMA (shape memory alloys) springs placed within cavities that longitudinally cross the cylindrical segments.

The cavities are not in the center of the cylinder but are displaced towards the outside segment. The springs are placed symmetrically at 120° within each segment, while their positions between two consecutive modules are out of phase-by-phase 40°.

The cavities have a diameter more significant than the outer diameter of the springs to allow their correct contraction movement from which they exert force. Integrating the springs within the segment allows for a more compact design.

**Figure 5:**
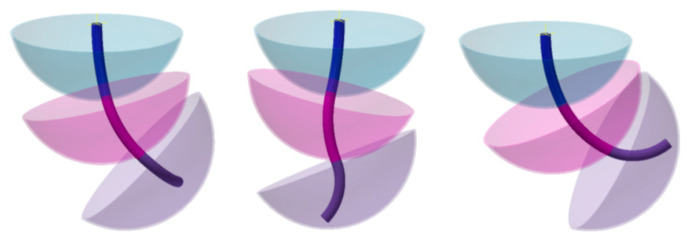
Range of motion of the three segments. Source: authors.

The individual or combined action of the springs allows the segment to bend around multiple axes. Thanks to this combination, each segment consists of three DOFs, totaling nine DOFs. A single segment operated in this way can position its end on a surface similar to a spherical cap. The radius of the sphere depends directly on the length of the segment. The three-segment composition expands the range of motion (Figure 5).

**Figure 6:**
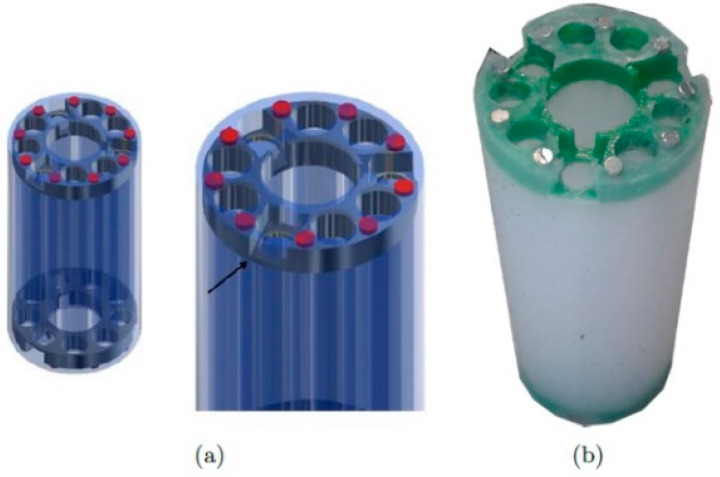
One of three silicone segments. (**a**) the CAD design, and (**b**) the actual segment. The red elements in the CAD represent the magnets, which are located in the green PLA disc. Source: authors.

The segments are connected with PLA 3D-printed discs embedded within the silicone body for a compact design. Small neodymium magnets are placed on these discs to allow quick and easy mounting and dismounting (Figure 6). The magnets have a diameter of 3 mm, a height of 2 mm, and an attraction force of 252 g. Nine of these magnets distributed on the contact surface between the segments ensure that the segments stick together during robot operation. In addition to allowing the segments’ junctions, the discs serve as an anchor point for the SMA springs.

The springs used have been custom-made for the robot to achieve the desired length and force parameters. A Nitinol wire with a diameter of even 0.5 mm has been used to manufacture them. The choice of this diameter represents the best compromise between the force necessary to actuate the robot (smaller diameters did not exert enough force) and the desired size for the robot (larger diameters would have meant a general increase in the size of the robot).

The thickness of the robot walls is 2 mm to achieve the maximum possible bending. The walls are the areas between the cavities and the outside of the silicone body.

**Figure 7:**
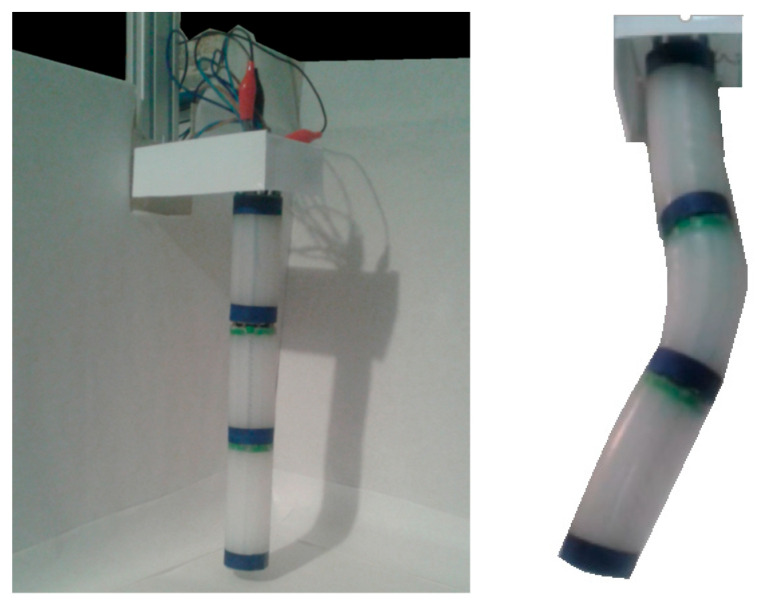
The final prototype. Source: authors.

Each spring is independently powered by MOSFET transistors controlled by a PWM signal from an Arduino UNO board. The SMAs are activated in the all-nothing mode so that each segment can be activated in the seven states derived from combining the binary states of the three actuators. It is impossible to activate all three simultaneously because the silicone is not compressible. In Figure 7, it is possible to see the complete robot.

### Vision System for Open Loop Control

**Figure 8:**
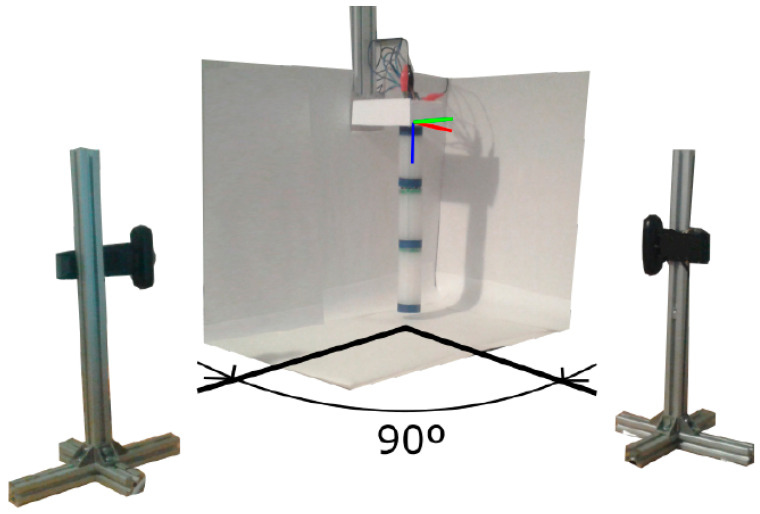
Vision system configuration. Source: authors.

A vision system capable of detecting the different positions of the segments of the robot and the final effector has been implemented to know if the movements made by the robot comply with the initial specifications. The idea of placing flex sensors on the outer walls of the robot was discarded so as not to add resistance to the body and thus hinder flex movement. The system consists of two cameras and circular marks surrounding the segments’ ends. Each camera captures the robot’s movement in the corresponding plane, as shown in Figure 8.

The data obtained from each camera is put together to obtain the position of the marks with respect to a reference system located in the upper part of the robot. These rings are perceived as rectangles in the images captured by the cameras. By treating the images, it is possible to determine the inclination of the axis of the rectangle and, therefore, the inclination of the end of the segment. The vision system has been implemented in MATLAB.

**Figure 9:**
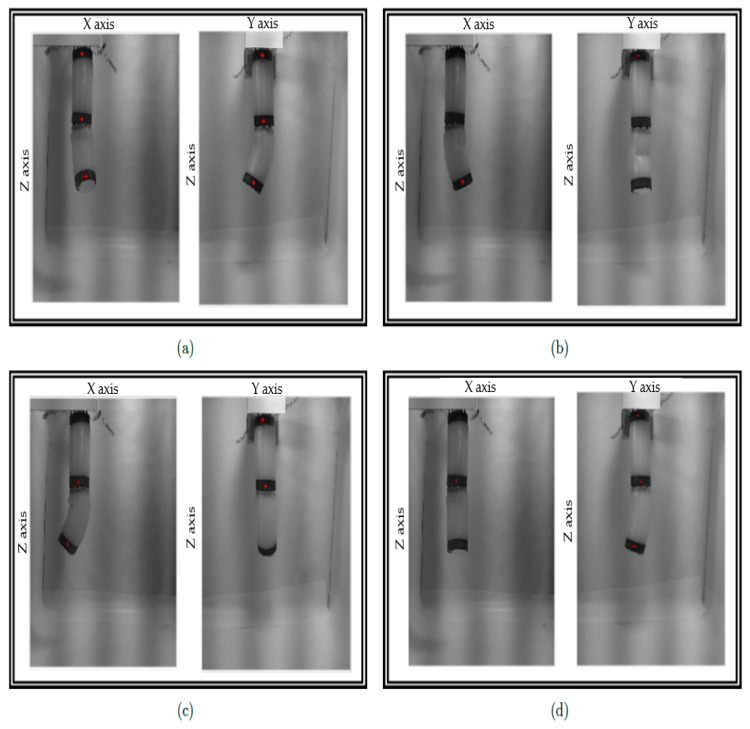
Experiments. The four pictures (**a**–**d**) show different positions reached by the soft robot. Source: authors.

The experiments have been carried out with only two of the three segments. Therefore, 49 different positions can be carried out. These positions result from combining the seven positions (counting the rest position) that the first segment can reach by activating its springs individually or not with the seven positions of the second segment. Figure 9 shows examples of the test performed.

**Figure 10:**
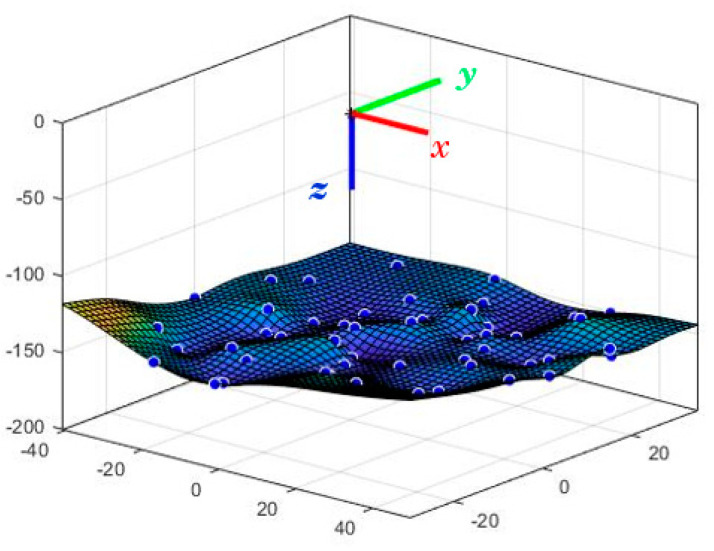
End effector positions. Source: authors.

From all the tests carried out, the different positions of the end effector are shown in the graph of Figure 10.

In the XY plane, the end effector can travel a maximum of 40 mm in the negative X direction and a maximum displacement of 55 mm in the positive direction. Regarding the *y*-axis, the displacement in the negative direction of the axis can reach 30 mm, while in the positive direction, it reaches 40 mm. Regarding the *z*-axis, establishing a minimum height of 160 mm corresponding to the robot’s rest position, the action of the springs can raise the final effector to 135 mm; that is, it can perform a displacement of 45 mm.

**Figure 11:**
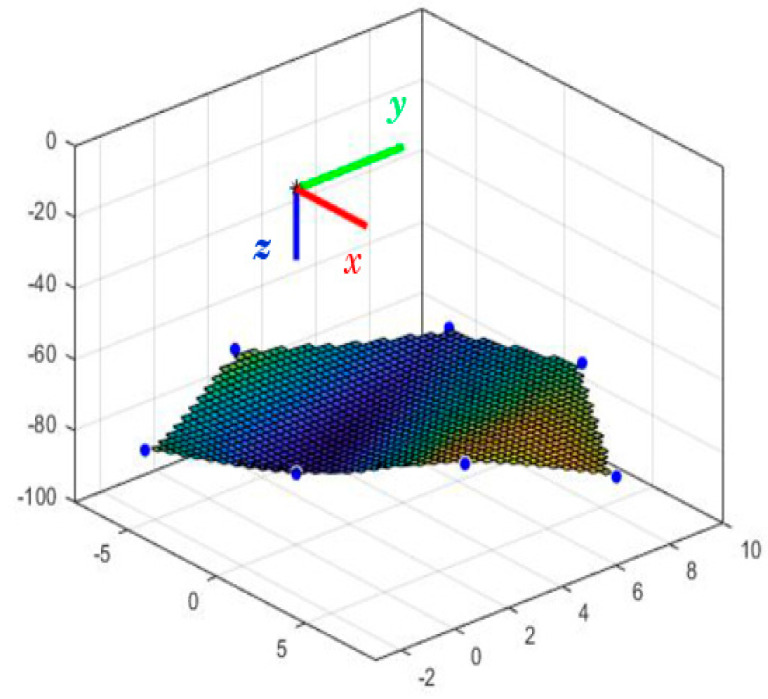
Positions of the end of the first segment. Source: authors.

Furthermore, Figure 11 shows the seven positions assumed by the trailing end of the first segment.

It is necessary to emphasize that the springs of the first segment must overcome the force necessary to bend this segment and lift the second segment. Because of this, the total displacement of the first segment is less than that of the robot’s end effector. It displaces −8 and 9 mm on the *x*-axis and −3 and 10 mm on the *y*-axis. In the case of the first segment, the lower limit on the *z*-axis is 80 mm, and the maximum displacement observed is 7 mm in the upward direction. In addition, the inclination that the first segment can reach is less than that of the second, presenting a maximum angle of 20 degrees. The end of the second segment reaches 35 degrees incline with the first segment at rest.

Once the positions are determined, a matrix is generated. It relates the positions of the end effector to the springs that must be actuated to move.

As graphs represent the range of motion, there is no range homogeneity in different directions. This is because the springs sometimes exert different forces. Added to this effect is the elastic behavior of the body, with folds able to appear that make the resulting position vary.

## 3. Modeling of the Robot with Finite Elements

In developing the kinematic model of continuous robots, the equation of motion given by continuum mechanics (2) cannot be solved since it involves an infinite number of degrees of freedom.

(3) $$\nabla \cdot \;\sigma +\rho f=\rho \;\left(\frac{\partial v}{\partial t}+v\cdot \nabla v\right)\;$$

Due to the high calculation times required by FEA, the model was first developed for a single segment. Once the results were satisfactory, the system was adapted for two concatenated robot segments. For the same reason, the geometry of the model was simplified. All FEM simulations were conducted with the ANSYS Mechanical.

**Figure 12:**
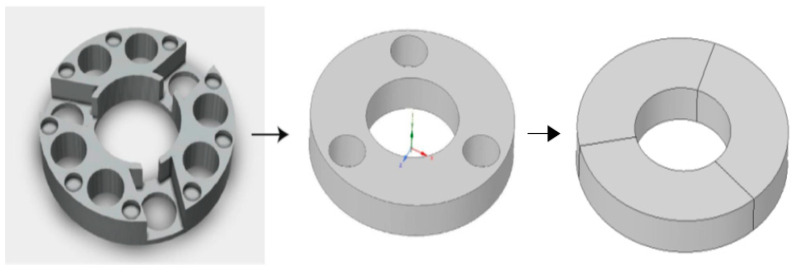
Simplifications in disk modeling. Source: authors.

The first simplification involves the disk, as shown in Figure 12.

**Figure 13:**
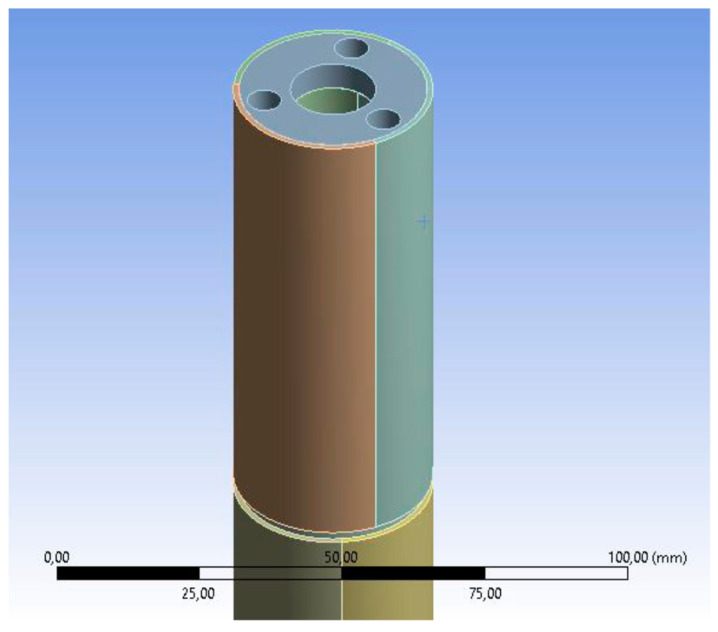
Simplification of the robot body model. Source: authors.

Furthermore, the disks and the robot body were divided into three equal parts to reduce computation time, as shown in Figure 13. The SMA springs were replaced by threads or tendons of standard material, which move vertically during activation. The threads were defined by generating 0.5 mm diameter lines centered on the sides of the longitudinal cavities of the silicone body.

**Figure 14:**
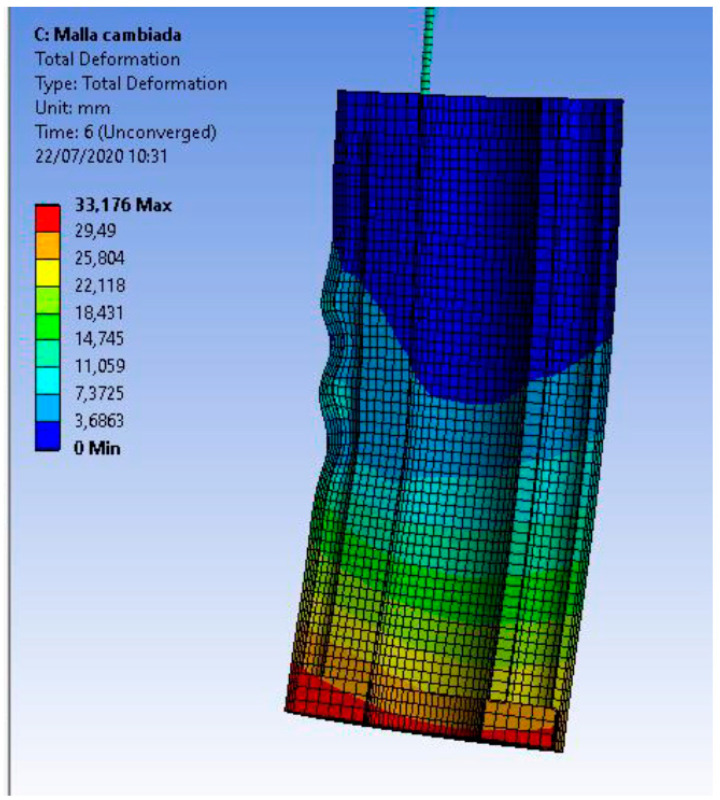
Simulation of the segment with folds. Source: authors.

The size of the finite elements considers the minimum number necessary to carry out an adequate simulation in a shorter calculation time. A significant number of elements in the silicone body increase computation time to unsustainable levels and generate the creases that the real robot exhibits by bending its segments, as shown in Figure 14. Although these folds are part of the behavior of the physical robot, they are unwanted since they affect its ability to move and converge.

The meshing of the threads significantly affects the lack of convergence since the number of elements in them affects their level of flexibility; the more elements the threads have, the more flexible they will be. In addition, as mentioned above, they must be flexible enough to conform to the curve of the silicone body after deformation; that is, it is intended that the threads bend together with the silicone body. However, many elements also negatively affect the interaction of the threads with the body. If the mesh of the threads is very thick, its elements turn out to be so small that they are ignored by the mesh of the silicone body passing through it at some points.

Finally, the type of elements used in the silicone geometry and the discs are Solid185.

**Figure 15:**
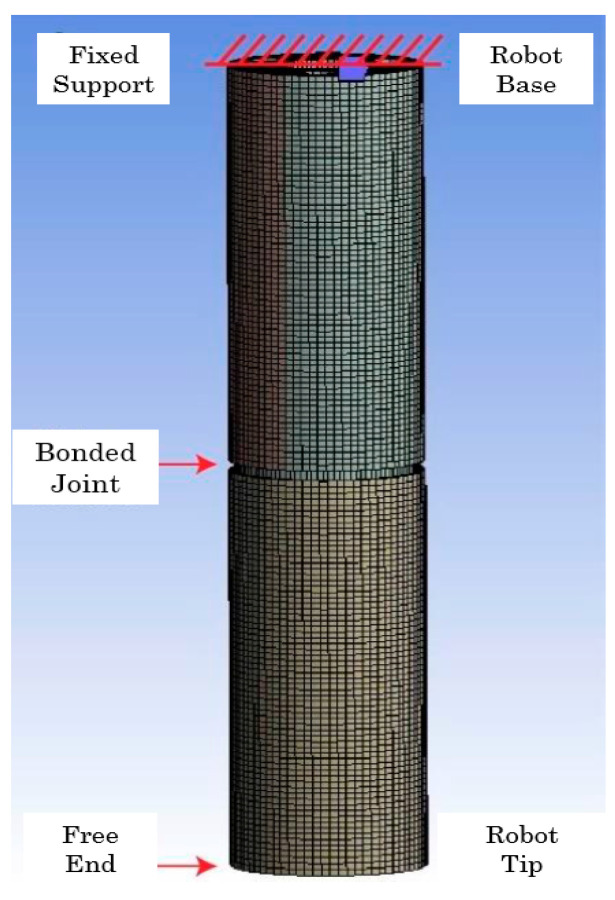
Boundary conditions in the simulation. Source: authors.

**Figure 16:**
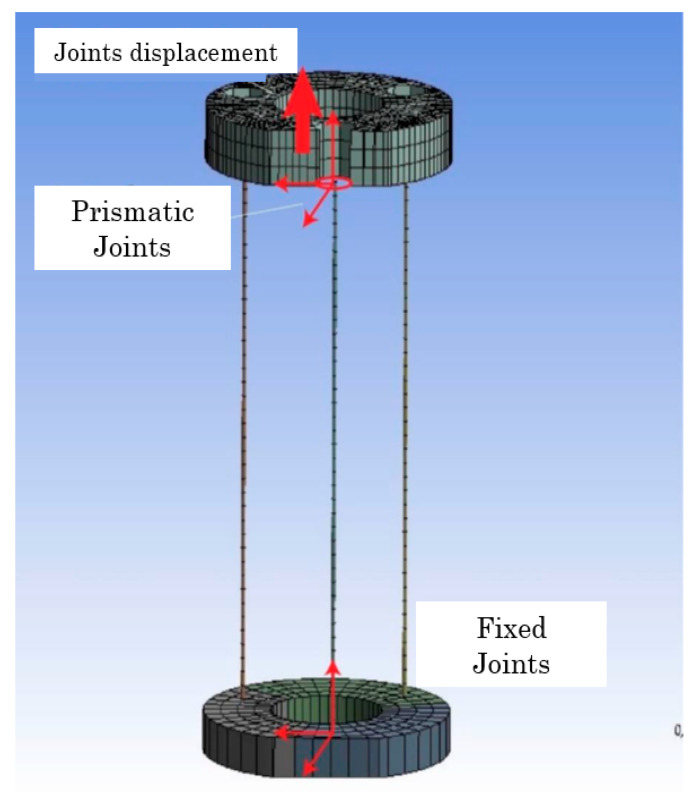
Articulations and displacements of the threads. Source: authors.

The model presents fixed support to the upper rigid disk of the first segment, that is, at the robot’s base. While the tip of the second segment is free, and the only added load is standard gravity. Both segments are joined by a “bonded” type joint. Each cable is connected to the robot segment through a joint at each end. The lower joint, which joins the cable to the lower rigid disk, is of the fixed type, while the upper joint, which “holds” the cable in place, is prismatic or translational; the latter ensures that the cable can only move vertically. Finally, the joint offsets are placed on top of the end of each cable (Figure 15 and Figure 16).

The different simulation steps determine which threads will be displaced and the value of the displacements. The threads are activated in pairs, each corresponding to a different letter. Table 1 shows Configuration A in the second segment.

For Configuration A of the second segment, threads 1 and 2 move 4 mm up while thread 3 does not move. At step 6, these displacements on leads 1 and 2 increase to 8mm while lead 3 remains at 0. This continues until leads 1 and 2 reach their maximum displacement to return to 0, the resting state.

These activation settings allow each segment to perform three different movements since our robot has two segments; can perform nine different moves. All possible combinations of these activations between the two segments are shown in Table 2.

The bending angle was calculated by defining a remote-control point on the lower disk of each segment and calculating its rotation in the X, Y, and Z directions. The results were compared with those obtained in the first phase of the work. The physical robot reached a maximum angle of approximately 20° at the tip of its first segment, while the second segment reached a maximum angle of approximately 35°.

**Figure 17:**
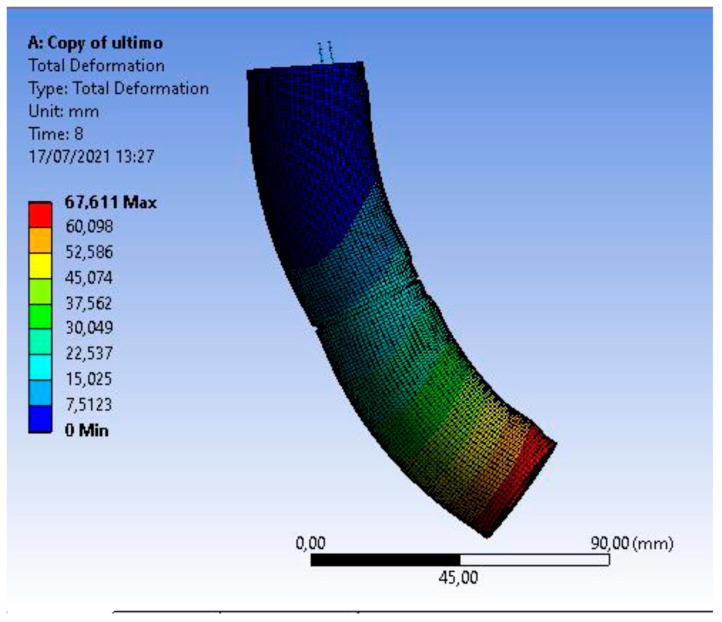
Deformation of the soft body of the robot under the action of the threads. Source: authors.

The tests showed that the required bending angle in the first segment is achieved with a vertical displacement in the threads of −12 mm. On the other hand, the required bending angle in the second section is reached with a displacement in the threads of −13 mm. Figure 17 represents the deformation of the soft body of the robot under the action of the wires.

**Figure 18:**
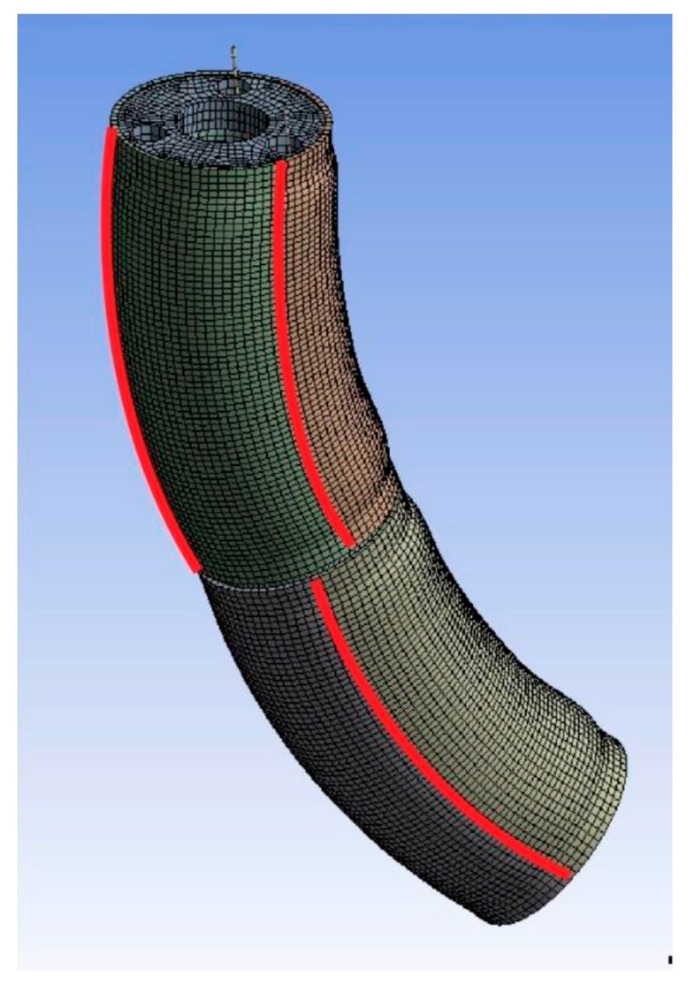
Deformed node paths. Source: authors.

Once the results were considered acceptable, the data was extracted. The extracted data define the curves and relate their deflection to the activation of the actuators. Consequently, three equidistant paths were defined along the outside of each silicone segment, as seen in Figure 18.

## 4. Implementation of the PCC and the Neural Network

The extracted data comprises the (1) displacements of threads in millimeters and the (2) displacements of rows of nodes after deformation. They are divided according to (1) the segment from which the data was extracted, (2) its simulation stage, and (3) its activation configuration.

Microsoft Excel was used as a tool to save and process the data. Spine curves were calculated for each segment and stage through the coordinate of the knots before and after deformation. To verify these spine curves, they were plotted in Matlab.

Matlab was also used to apply the PCC method and calculate the parameters *K*, ∅, *S* at each Step. A function called “arcParameters” has been developed that includes all the necessary calculations.

The arcParameters function takes the configuration of the first segment, the Step, the backbone of the first segment, the backbone of the second segment, and finally, the offsets of the threads. It returns the arc parameters for the first and second segments. In addition, it generates an Excel table where the calculated data is saved and organized for later use in the neural network.

Multilayer perceptrons or MLPs were chosen for the neural network as they have been successfully applied to learn forward and inverse kinematic models of robots.

MLPs are feedforward neural networks with a minimum number of 3 layers composed of nodes (the input layer, the output layer, and at least one hidden layer). Each node in one layer is connected to all nodes in the next layer through a weight *wji*. Furthermore, all nodes except those belonging to the input layer have an activation function. The activation or step function takes the output of the previous node and acts as a filter or limiting function, modifying the value of the result. The learning process consists of calculating the error of the final output of the network to, based on it, update all the weights *wji*. These weights can be updated by backpropagation of the error *δyi* between the desired value and the actual output of any neuron.

Matlab provides a tool (Machine Learning) that, through the function feedforwardnet(hiddenSizes, trainFcn), generates an MLP with the desired hidden layers and the training function. It also provides functions to train the network based on an input dataset and the desired output dataset.

**Figure 19:**
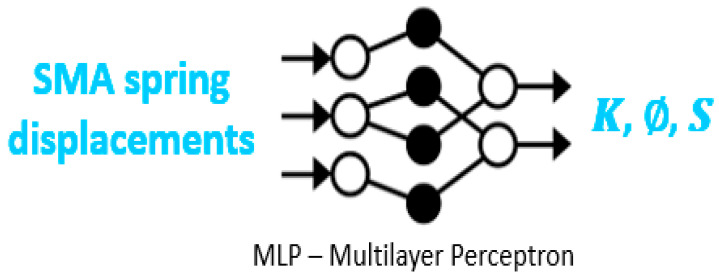
Scheme of the neural network. Source: authors.

In this case, the input data set consists of the SMA spring displacements in mm, while the output data set consists of the arc parameters *K*, ∅, *S* as shown in Figure 19.

The number of hidden layers was decided considering the results presented in the work of Kovandžić [23], where it was shown that a multilayer network compared to a single hidden layer network did not present a significant improvement. For this reason, we decided to use six hidden layers in our network.

Another important aspect is that the neural network can only approximate continuous functions; however, the rotation angle ∅ is not continuous because, from 0°, it jumps to 360°. For this reason, it was decided to replace the value of ∅ with sin ∅ and cos ∅.

The training dataset saved in an external Excel table was partitioned between the input array and the expected output array. However, the first tests gave erroneous results because specific parameters of the first segment interfered with the parameters of the second segment. These problematic parameters are those that are taken during the resting state of the segments. Since they do not present a curve because there is no activation of the actuators, the function arcParameters; therefore, it produces calculation errors in these specific situations.

The chosen solution to this problem, being the simplest, was to use two different neural networks, one for each segment, and ignore the data that contains zero displacements in the threads. Although the position of the second segment depends on the movements made by the first segment, the parameters of the arc, now being *K*, sin ∅, cos ∅, *S*, only describe the configuration space and not the position in the workspace.

Although these changes represent a considerable reduction in the training data, the results obtained were satisfactory in several tests.

**Figure 20:**
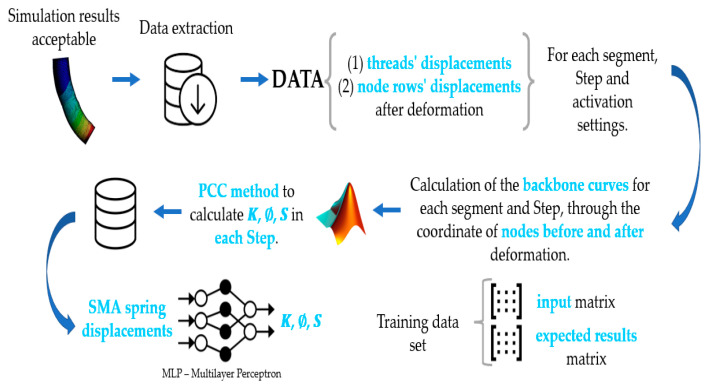
Scheme of the different phases that lead from the FEA simulation to neural network training. Source: authors [24].

A summary of the procedure described in Section 4 is shown in Figure 20.

## 5. Results

The results shown below can be considered divided into two sections. In the first part, the results obtained in the simulations are compared with the data obtained from the real robot through the vision system to validate the finite element model used. In the second part, the results obtained by the neural network are compared with those calculated in the simulation.

The work carried out was validated by comparisons with previous data from the vision system, as shown in Table 3.

The minimum displacement value in the *z*-axis is equal to the rest position of the tip of the free segment. The Z-coordinate at rest from the tip of the free end will be 160–162 mm. At the same time, the maximum value is equal to the displacement in the direction toward the origin since the robot will constantly flex upwards. The displacement value can be obtained by subtracting the idle value minus the maximum value.

The most considerable discrepancies observed in Table 4 are given by the difference between the orientation of the physical robot and the orientation of the robot model in the simulation. However, the values are pretty close considering the absolute values of the displacements.

As shown in Table 4, the free end of the physical robot can move a distance in the X plane of 95 mm in total, while the simulation shows a displacement of 92.58 mm in total. Regarding the displacement in the *y*-axis, the asymmetry of the physical robot affects the results. In the case of a perfectly constructed robot, the displacement in the *y*-axis would be almost equal to the displacement in the *x*-axis, as the simulation data show.

Therefore, it is considered that the other errors are within acceptable ranges, thus concluding that the simulation represents the behavior of the flexible robot with a good level of fidelity.

After the training phase, tests were carried out to verify the accuracy of the approaches, with some data reserved for these tests. Table 5 and Table 6 show the comparison between the expected and obtained values.

These results show that, while some parameters are almost exactly close, others have a somewhat considerable deviation, as is the case of sine and cosine.

The methodology was first tested considering a single segment of the robot. In that case, the neural network presented better results. However, as explained in Section 4, training data was considerably reduced in the case of two segments to avoid errors generated from the data that contains zero displacements in the threads. This decrease in available data entails less training of the neural network. For this reason, not all parameters fit exactly the attended values.

A possible solution to provide more data to the neural network is to divide the thread displacements in ANSYS among more steps and develop an application that automatically extracts this data.

## 6. Conclusions

The model of the robot was made with finite elements to achieve a model as similar as possible to the real system.

It was possible to simulate the nonlinear behavior of Ruǎn as a single segment and as a set of two segments, thus providing a data source to relate the behavior of the actuators with the bends that the silicone body presents.

The results of the simulations were compared with experimental data, verifying the accuracy of the simulated model. Once this model was validated, it was used, obtaining data sets that related the displacements of the actuators and the consequent displacement of the nodes corresponding to each module of the robot. The positions of the nodes were used to calculate the central line of the module (“backbone curve”). Finally, assuming for each module the PCC constant curvature hypothesis, the curve parameters were obtained and thus the final location of the end of the robot. A subset of these data (actuator displacement vs. PCC parameters) was used to train a neural network. The rest of the data obtained from the finite element model was used to validate the neural network.

The MLP neural network approximates nonlinear functions but requires a large amount of data to obtain results with an acceptable margin of error. Therefore, the development of an application that automatically extracts the data from the finite element simulation in an effective way is necessary.

This strategy has enabled fast, energy-efficient direct kinematic modeling through finite element simulations with machine learning tools.

## Figures and Tables

**Table 1:** Displacement of the threads. Configuration A. Source: authors.

Second Segment Activation				
Steps (s)	Thread 1	Thread 2	Thread 3	Configuration
5	4	4	0	A
6	8	8	0	
7	12	12	0	
8	13	13	0	
9	0	0	0	

**Table 2:** All possible activation combinations. Source: authors.

Segment 1		Segment 2		
Config.	Activated Threads	Config.	Activated Threads	
A	Thread 1—Thread 2	A	Thread 1	Thread 2
		B	Thread 2	Thread 3
		C	Thread 3	Thread 1
B	Thread 2—Thread 3	A	Thread 1	Thread 2
		B	Thread 2	Thread 3
		C	Thread 3	Thread 1
C	Thread 3—Thread 1	A	Thread 1	Thread 2
		B	Thread 2	Thread 3
		C	Thread 3	Thread 1

**Table 3:** Comparison of the displacement ranges between the second segment of the flexible robot and the simulation. Source: authors.

In-Plane Displacement Ranges (mm)						
	X		Y		Z	
	Max	Min	Max	Min	Max	Min
Real robot	55	−40	40	−30	135	160
Simulation	35.88	−56.7	52.48	−45.79	131	162

**Table 4:** Total displacement of the free end of the robot. Source: authors.

Total Displacement Capacity (mm)			
	X	Y	Z
Real Robot	95	70	25
Simulation	92.58	98.27	31

**Table 5:** Comparison of the neural network results for the first segment. Source: authors.

First Segment				
	*S*	sin ∅	cos ∅	*K*
Expected data	76.0837	0.8656	0.5008	0.0055
Data obtained	73.3643	0.9612	0.2007	0.0029

**Table 6:** Comparison of the neural network results for the second segment. Source: authors.

Second Segment				
	*S*	sin ∅	cos ∅	*K*
Expected data	74.1405	0.8649	0.5020	0.0080
Data obtained	74.3726	−0.2829	0.2421	0.0070

## References

[1] Chen X., Zhang X., Huang Y., Cao L., Liu J. (2022). A review of soft manipulator research, applications, and opportunities. J. F. Robot..

[2] Xavier M.S., Fleming A.J., Yong Y.K. (2021). Finite element modeling of soft fluidic actuators: Overview and recent developments. Adv. Intell. Syst..

[3] Cerrillo D., Barrientos A., Del Cerro J. (2022). Kinematic Modelling for Hyper-Redundant Robots—A Structured Guide. Mathematics.

[4] Webster R.J., Jones B.A. (2010). Design and kinematic modeling of constant curvature continuum robots: A review. Int. J. Rob. Res..

[5] Bieze T.M., Largilliere F., Kruszewski A., Zhang Z., Merzouki R., Duriez C. (2018). Finite element method-based kinematics and closed-loop control of soft, continuum manipulators. Soft Robot..

[6] Tawk C., Spinks G.M., in het Panhuis M., Alici G. (2019). 3D Printable Linear Soft Vacuum Actuators: Their Modeling, Performance Quantification and Application in Soft Robotic Systems. IEEE/ASME Trans. Mechatron..

[7] Elsayed Y., Vincensi A., Lekakou C., Geng T., Saaj C.M., Ranzani T., Cianchetti M., Menciassi A. (2014). Finite element analysis and design optimization of a pneumatically actuating silicone module for robotic surgery applications. Soft Robot..

[8] Kim D., Kim S.-H., Kim T., Kang B.B., Lee M., Park W., Ku S., Kim D., Kwon J., Lee H. (2021). Review of machine learning methods in soft robotics. PLoS ONE.

[9] Chin K., Hellebrekers T., Majidi C. (2020). Machine learning for soft robotic sensing and control. Adv. Intell. Syst..

[10] Runge G., Wiese M., Günther L., Raatz A. A framework for the kinematic modeling of soft material robots combining finite element analysis and piecewise constant curvature kinematics. Proceedings of the 2017 3rd International Conference on Control, Automation and Robotics (ICCAR).

[11] Spong Mark W., Seth H., Vidyasagar M. (2006). Robot Modeling and Control.

[12] Ganji Y., Janabi-Sharifi F. (2009). Catheter kinematics for intracardiac navigation. IEEE Trans. Biomed. Eng..

[13] Hannan M.W., Walker I.D. (2003). Kinematics and the implementation of an elephant’s trunk manipulator and other continuum style robots. J. Robot. Syst..

[14] Joie-La Marle C., Parmentier F., Coltel M., Lubart T., Borteyrou X. (2022). A Systematic Review of Soft Skills Taxonomies: Descriptive and Conceptual Work. PsyArXiv.

[15] Chirikjian G.S., Burdick J.W. (1995). Kinematically optimal hyper-redundant manipulator configurations. IEEE Trans. Robot. Autom..

[16] Tatlicioglu E., Walker I.D., Dawson D.M. Dynamic modelling for planar extensible continuum robot manipulators. Proceedings of the 2007 IEEE International Conference on Robotics and Automation.

[17] Murray R.M., Li Z., Sastry S.S. (2017). A Mathematical Introduction to Robotic Manipulation.

[18] Ivanescu M., Popescu N., Popescu D. A variable length tentacle manipulator control system. Proceedings of the 2005 IEEE International Conference on Robotics and Automation.

[19] Falkenhahn V., Hildebrandt A., Neumann R., Sawodny O. Model-based feedforward position control of constant curvature continuum robots using feedback linearization. Proceedings of the 2015 IEEE International Conference on Robotics and Automation (ICRA).

[20] Della Santina C., Katzschmann R.K., Biechi A., Rus D. Dynamic control of soft robots interacting with the environment. Proceedings of the 2018 IEEE International Conference on Soft Robotics (RoboSoft).

[21] Hannan M.W., Walker I.D. (2000). Novel kinematics for continuum robots. Advances in Robot Kinematics.

[22] Jones B.A., Walker I.D. (2006). Kinematics for multisection continuum robots. IEEE Trans. Robot..

[23] Kovandžić M., Nikolić V., Simonović M., Ćirić I., Al-Noori A. (2019). Soft Robot Positioning using Artificial Neural Network. Facta Univ. Ser. Autom. Control Robot..

[24] Terrile S. (2022). Soft Robotics: Applications, Design and Control. Ph.D. Thesis.

